# Skirts as Flags: Transitional Justice, Gender and Everyday Nationalism in Kosovo

**DOI:** 10.1111/nana.12593

**Published:** 2020-03-13

**Authors:** Vjollca Krasniqi, Ivor Sokolić, Denisa Kostovicova

**Affiliations:** ^1^ Faculty of Philosophy University of Prishtina Kosovo; ^2^ European Institute London School of Economics and Political Science London UK

**Keywords:** art, gender, nationalism, transitional justice, Kosovo

## 1 INTRODUCTION

Wartime rape is ubiquitous in contemporary conflicts. Its commission is deeply implicated in the gendered notions of a nation: a woman's body is constructed as a target in a conflict that involves different identity groups and as an object of protection within a nation. Addressing past wrongs in view of repairing relationships after human rights violations across the identity divide is an integral part of post‐conflict recovery (Murphy, 2017, pp. 22–23). The practice of transitional justice also has a “constitutive” effect on the building of the new democratic order and peace (Teitel, 2000). Post‐conflict justice provides an opportunity for reordering gender relations within post‐conflict nation building. This process requires the recognition of both a victim‐ and a gender‐specific harm, such as wartime rape. However, the practice of transitional justice often perpetuates “transitional *in* justice” (Loyle & Davenport, 2016), of which gender injustice is a part.

In this article, we bring the perspective of everyday nationalism to the feminist theorizing in the field of transitional justice and investigate gendered dimensions of post‐conflict nation building. Our aim is to understand possibilities for achieving gender‐just peace characterized by the transformation of gender relations, as well as their obstacles. Feminist scholarship has captured complex, contested, and ambiguous dynamics of shifting gender relations in conflict and post‐conflict settings in the everyday domain. Despite increasing understanding of women's agency and its limits, the entrenchment of dominant hierarchical norms at the intersection of gender and the nation remains puzzling. Everyday nationalism directs attention to mundane aspects of nationhood. It also offers a bottom–up perspective on top–down processes of “formal” nationalism and their interplay with everyday constructions of nationhood. The alignment between these bottom–up and top–down processes reveals how national ideologies are legitimized and hierarchical gender relations entrenched. We ask, does the public recognition of wartime sexual violence and women's suffering challenge the norms and habits of masculine nationhood and pave the way for a new start free of patriarchal hierarchies? Or does it entrench a gendered war “metanarrative” (Björkdahl & Mannergren Selimovic, 2015, p. 172) and with it, unequal gender relations?

We study a public art installation about wartime sexual violence in Kosovo aimed at tackling the stigma and silence about wartime rape. The analysis is focused on how this artistic practice, as a symbol, discourse, and performance, as well as an intervention in the everyday domain, offers recognition of wartime sexual violence, and how this recognition responds to, or interacts with, existing gendered dynamics of nationhood. Drawing on Malešević (2013, p. 14), we argue that nationalism and nationhood transcend the public/private dichotomy by connecting institutions and organizations, such as public art installations, to everyday microinteractions. We show that the public endorsement of the art project and the acceptance of wartime sexual violence result in the recognition of the war crime but not the victim. Dynamics of everyday nationalism reinforce gender asymmetries and women's marginalization in a nation‐building process even while their suffering is being acknowledged publicly.

Twenty years after the war in Kosovo ended, justice for ethnic Albanian women victims of sexual violence is still largely elusive. Their suffering has been sidelined both in international criminal prosecutions as well as in hybrid domestic war crime trials. The recent adoption by Kosovo's parliament of a reparations law for wartime sexual and gender‐based violence marks formal progress. But, its impact on actual redress for this wartime harm has been limited. One of the major obstacles for women coming forward to claim the reparations is the stigma surrounding wartime sexual violence. The stigma is steeped in gendered patriarchal mores playing themselves out in the politics of postwar peacebuilding within the victims' national community, and it pervades everyday life.

By focusing on how an artistic intervention can promote justice for victims of wartime rape, we explore an avenue for supporting gender‐just peacebuilding that is an alternative to women's activism, legal responses, and formal gender equality policies. Despite the “context‐specific natures of claims of justice” (Murphy, 2017, p. 6), the case study of Kosovo reflects the typical pattern of gender‐based harm and the challenges of building gender‐just peace after a civil war. Therefore, our findings reveal everyday dynamics of gendering nation building and contribute to the wider understanding of how the redress for wartime sexual violence perpetuates gender‐insensitive peace (Chinkin & Kaldor, 2013).

Empirical research in this article draws on a range of sources. These include the analysis of the *Thinking of You* art installation, published interviews with the artist, reports of domestic and international media outlets (in Albanian and English), a documentary film about the installation with the same title (*Mendoj Për Ty|Thinking of You*–Documentary), and speeches by former president of Kosovo, Atifete Jahjaga. We first outline feminist perspectives on transitional justice and present the analytical gains of applying an everyday nationalism perspective to the study of gendered construction of nationhood. This is followed by a background section on the war, sexual and gender‐based violence, and postwar stigma in Kosovo, as well as an overview of the art installation. The analysis is organized around three conceptual dimensions of everyday nationalism: symbols, discourse, and performance.

## 2 GENDER AND TRANSITIONAL JUSTICE

Feminist attention to gender in transitional justice is a relatively new phenomenon (Nì Aoláin, 2012; Bell & O'Rourke, 2007; Baines, 2011). It is characterized, first and foremost, “by centralising gender in the analysis of the traditional concerns of transitional justice mechanisms” (Gyimah, 2009, pp. 20–21). As such, it represents a response to the previous neglect of women's experiences during and after the conflict (Simic, 2016a, p. 2). Scholars have shown that the nature of wartime violence against women is related to gendered constructions of a nation. The practice of post‐conflict transitional justice in response to the legacy of sexual and gender‐based violence provides an opportunity to address this gendered logic and these consequences of conflict. But it also has wider implications for how a nation is constructed after a conflict, including the women's place therein.

As a war crime, sexual and gender‐based violence reflects the gendered notion of the nation constructed through comparisons of a nation with a family, in which men and women have “natural” gender‐specific roles (Anderson, 1991, p. 7; McClintock, 1991; Nagel, 1998; Skurski, 1994; Yuval‐Davis, 1997; Žarkov, 2007). Conceived as metaphors, nation‐as‐woman and woman‐as‐nation become everyday battlegrounds of actual group struggles (Peterson, 1999, p. 47). The land, or body, and honour of the passive, feminized nation must be defended by males (Helms, 2013; Massad, 1995; Mosse, 1985; Peterson, 1999). Ethnosexual transgression, such as rape of one's homeland or women, is one of the most potent justifications for military intervention against ethnic “Others” (Nagel, 2003, p. 256). In sum, land and history, as Sofos (1996, p. 87) points out, “can be and are conquered over women's bodies.” The end of a conflict and, in particular, the practice of transitional justice that recognizes gender‐specific wartime violence are indicative of how gender relations within nation building are (re)constructed during peacebuilding.

As with the nature of violence in conflict, the practice of transitional justice has also been revealed to be gendered. The omission of wartime sexual violence from transitional justice processes, whether retributive, such as international and domestic war crime trials or restorative, such as truth commissions, has resulted in a reckoning with past wrongs that has marginalized women's experiences of conflict (Kashyap, 2009; Nagy, 2008; Oosterveld, 2009). At the same time, in their lived environments, women often remain silent about wartime rape because of a risk and fear of stigmatization, ostracism, and even physical punishment by members of their own communities. Such stigmatization persists owing to normative, structural, and political factors (Graybill, 2001; Krog, 2001; Simic, 2016b). As a consequence, women's suffering is unacknowledged, impacting the prospects of redress for injustice—despite calls for a subtler understanding of silence as an agential act that is empowering (Porter, 2012, p. 35; Mannergren Selimovic, 2018).

Speaking out about gender‐based violence can also have a mixed outcome. The assertion of women's agency through advocacy about wartime rape, which may also include public testimony about their suffering, has resulted in normative and policy shifts at international and national levels (Patterson‐Markowitz, Oglesby, & Marston, 2012). At the same time, it has also created different gendered dynamics. The sole focus on sexual violence has reduced a range of women's experiences of conflict to one dimension of harm endured by women (Heineman, 2011; Tabak, 2011; Turano, 2011), overlooking many different forms of victimhood such as sacrificing mothers of soldiers or being engaged as fighters (Cockburn, 1998; Griffin & Braidotti, 2002; Reilly, 2010, p. 239; Shadmi, 2000). Furthermore, gendered transitional justice, as a theory and practice, has prioritized analytically women in relation to men victims of sexual violence (Simic, 2016a).

In accounting for “gendered peace” (Pankhurst, 2008), scholars have focused on the practice of transitional justice through a range of transitional justice mechanisms, as well as the contents of peace agreements and their gender‐responsive provisions (Ellerby, 2016) and formal policies pursued by post‐conflict states that instrumentalize justice for wartime rape (Loken, Lake, & Cronin‐Furman, 2018). The understanding of the constraints on justice seeking constituted by everyday enactment of nationhood in a post‐conflict environment remains a lacuna in this scholarship. We address this gap by heeding the need identified by Ní Aoláin (2012) to return the gaze to the cultural, material, and geopolitical sites where transitional justice is practiced. The theoretical perspective of everyday nationalism allows us to reveal how transitional justice is implicated in reproducing gender hierarchies in post‐conflict nation building.

## 3 ENGENDERING EVERYDAY NATIONALISM

Everyday nationalism provides an analytical lens through which to examine gender relations in nation building and their reordering, which may be messy and contingent. Everyday nationalism is a subfield in the study of nationalism that focuses on ordinary people and their agency in order to better understand the lived experience of nationalism (Knott, 2015, p. 1). It posits ordinary individuals as the co‐constituents, participants, and consumers of national symbols, rituals, and identities (Knott, 2015). The concept enables us to investigate empirically how nationalism is constructed from below by examining ordinary relations, individuals, settings, and items. Scholars of the field examine how the nation is reproduced by “ordinary people doing ordinary things in their ordinary lives” (Fox, 2018, p. 862).

Everyday nationalism is derived from Billig's banal nationalism, although the exact relationship between the two concepts is much debated (including a symposium on the topic in this journal^1^). Banal nationalism is an understanding of nationalism as a widespread ideology diffused to everyday experience, which results in the unconscious reproduction of the nation by citizens (Duchesne, 2018 p. 844). It reveals the taken for grantedness of nationhood, which it opens for analysis. Billig's approach regards nationalism exclusively as an ideology that can often unknowingly be reproduced by ordinary citizens (Duchesne, 844; Billig, 1995). Banal nationalism is, therefore, seen as inherently negative, and this prescribed normative dimension arguably restricts its analytical purchase. It overlooks broader political, social, and cultural effects of a variety of symbols, discourses, and performances of nationalism. Some of these symbols, discourses, and performances, as we show in our analysis in the case of justice for wartime sexual and gender‐based violence, can advance gender‐just peace that acknowledges gender‐specific harm suffered by women. Applying everyday nationalism to the study of gender relations in nation building helps us understand the role of ordinary people and their agency while taking into account the complex and contingent nature of gender and nationalism (Brubaker, Feischmidt, Fox, & Grancea, 2006; Fox & Miller‐Idriss, 2008). Gender and the nation are both spoken, chosen, performed, and consumed (Fox & Miller‐Idriss, 2008). Both are constructed and unstable categories that are continuously performed and reproduced (Peterson, 1999, p. 55). Gender relations in the domain of the everyday have been extensively examined by anthropologists. Immersion in women's daily lives as a method of scholarly inquiry of gender relations within a nation has revealed the difficulty, complexity, and ambiguity involved in the assertion of women's agency and recognition of their subjectivity. Helms (2013) has shown how Bosnian women activists obtained respect and standing by using essentialist representations of women strategically. New conflicts, which are a consequence of adjusting gender relations, have been exposed, along with their impact on relations between men and women, as well as between women. Aretxaga (1997) recounts how republican women's “Dirty Protest” in the Armagh women's prison in Northern Ireland in 1980 and 1981 addressed the marginalization of women's contribution to the national struggle by using the potent symbolism of menstrual blood, but the protest also caused a fissure in the feminist movement in Ireland. Anthropologists have captured multilayered processes of renegotiations of gender relations, having rejected linear accounts of assertion of women's agency, as, for example, Mookharjee (2015) did when depicting simultaneous processes of shaming and celebration of “birangonas”—the women who suffered gender‐based violence in the 1971 Bangladesh war. Although revealing about women's daily struggles for equality within a political space of a nation, this scholarship has not engaged directly with theories of nations and nationalism. Rather, they have used nations and nationalism as concepts through which the feminist analysis of gendered structures, norms, and ideologies are refracted. In our analysis, we reverse the analytical gaze directed at gender relations. Underpinned by feminist theories that conceptualize gender “as a lived social relation; a location that is structural, symbolic and discursive” (McNay, 2004, p. 175), we apply the everyday nationalism perspective to reveal interactions in the realm of the everyday that reinforce subordinate subjectivities through the alignment of bottom–up and top–down constructions of the nation.

The expression and contestation of the nation's symbols and discourses in everyday life is an integral part of such investigation (Goode & Stroup, 2015, p. 723). Ethnographic methods have been the most common way of studying everyday nationalism because they allow for a rich observation of the practices of everyday life and capture the lived experience of nationalism (Goode & Stroup, 2015; Hearn, 2007; Knott, 2015). But, as we demonstrate in our analysis, ethnography can also be limited by its microanalytical approach and easily overlooks and downplays the impact and interaction of processes both at the formal and at the informal level (Smith, 2008, p. 567). As Malešević (2013, p. 130) argues, institutional frameworks can come to constitute the intimate world of ordinary individuals, constraining their agency. Consequently, the analysis of the “active construction” of nationalism (Mann & Fenton, 2009, p. 518) in a “naturally occurring” setting of the mundane also ought to investigate the complex dialectic between top–down and bottom–up processes and agents (De Cillia, Reisigl, & Wodak, 1999, p. 153). We can, therefore, simultaneously analyze “formal” nationalism, connected to the nation‐state; and “informal” nationalism, associated with collective events, civil society, and ritual celebrations (Eriksen, 1993). Approaching this interplay from the everyday perspective, we reveal how nationalism becomes entrenched. The approach speaks to an identified gap in the everyday nationalism literature, that of the relationship between nationalism, domestic political regimes, and legitimacy (Goode & Stroup, 2015, p. 721).

We draw on Malešević's (2013) theoretical framework to show that the top–down and bottom–up are mutually constitutive. Mundane, everyday nationalism could not exist without the elaborate institutions and organizations of “official” nationalism (Malešević, 2013, p. 131). Nationalism's vitality is based on the cultivation of an ideal institutional context, premised on long‐term organization and ideological build‐up, which allow a mundane and habitual sense of attachment to a nation to proliferate (Malešević, 2013, p. 140). Expressions of nationalism at the everyday level are, therefore, expressions of attachment to a specific social organization, such as the nation‐state (Malešević, 2013). From this perspective, the art installation is an institutional site where nationalism can be reproduced at the everyday level (Malešević, 2013, pp. 124–125).

Within such a framework, ordinary people are agents that reproduce the nation and nationalism, while their microinteractional lifeworld is influenced by top–down institutions. Individuals and institutions perform, consume, and frame nationhood in different ways, but both place structural limits to what or how widely they can choose (Malešević, 2013, p. 130). Nationalism can become both powerful and taken for granted only if it penetrates the microworld of family, friendships, neighborhoods, local communities, and kinship networks (Malešević, 2013, p. 151). In short, the top–down and bottom–up are tightly woven.

Ultimately, the framework of everyday nationalism allows us to glean a different understanding of the obstacles to justice for victims of wartime sexual violence by analyzing gender relations within social processes of nation building. Essentialist representations of women in nation building can limit women's agency, but they can also provide opportunities for engagement and action in the “male” public sphere, as Helms (2013) and Peteet (1991) have shown. They open identifiable opportunities for women to mobilize and act within nationalist constructs, which give their efforts legitimacy and ultimately counter gender norms (Fox, 1996). How these processes unfold and with what effect can be studied by analyzing art as a political and creative practice and tracing how gender and the nation intersect through symbols, discourse, and performance. The following section provides the context for studying these dynamics from the everyday nationalism perspective with an overview of the Kosovo conflict, the pattern of sexual and gender‐based violence, and the challenges encountered by women victims of wartime rape.

## 4 KOSOVO: WAR, RAPE, AND STIGMA

Kosovo was a self‐governing autonomous province within Serbia in the former Yugoslavia but without the same rights as the republics: Serbia, Montenegro, Slovenia, Croatia, Bosnia and Herzegovina, and Macedonia. In the late 1980s and 1990s, Serbian leader Slobodan Milošević incited ethnic nationalism in Serbia, underpinned by the ideology of controlling all “Serbian lands.” This nationalism was centred on Kosovo, the mythical cradle of Serbian nationhood. The policy of all‐Serb unification led to bloodshed in Croatia and Bosnia and Herzegovina. Meanwhile, repressive rule that privileged the Serb minority and oppressed the Albanian majority was instituted in Kosovo. Albanians mounted peaceful resistance that lasted for nearly a decade (Kostovicova, 2005). The worsening human rights situation in Kosovo, combined with a lack of international support for the Albanian nonviolent movement advocating Kosovo's independence, led to the emergence of violent resistance by the Kosovo Liberation Army in the late 1990s (Judah, 2000, pp. 135–197).

The Kosovo war broke out in the autumn of 1998. Serbian security and paramilitary forces mounted an ethnic‐cleansing operation against Albanians in Kosovo, prompting NATO to intervene militarily in the spring of 1999 to stop the violence. Sexual and gender‐based violence by Serbian forces was pervasive during the Kosovo war. Albanian women and girls of all ages were victims of sexual assault in their homes and local communities. Men were as well, although to a lesser extent. Rape camps were also established throughout Kosovo. Estimates about the number of women affected range between 10,000 and 40,000 (Farnsworth, 2008, pp. 13–14). However, these figures remain uncertain. This is in part because of Albanian women's reluctance to talk publicly about their suffering due to social isolation and stigma.

The NATO intervention lasted for 78 days until June 12, 1999. It ended the Serbian rule in Kosovo, but Kosovo did not gain independence. The province became a protectorate, administered by the United Nations and other international institutions: the European Union (EU), the Organisation for Security and Co‐operation in Europe (OSCE), and NATO from 1999 until 2008. The Albanian quest for Kosovo's independence continued. In November 2005, the UN Secretary General, Kofi Annan, appointed Martti Ahtisaari, the former president of Finland, as his special envoy to oversee negotiations between Kosovo Albanians and the Serbian government. The effort failed to produce an agreement: while the Albanians insisted on independence for Kosovo, the Serbs demanded that it should remain within Serbia. Following the failure of the negotiations, a UN proposal known as the Ahtisaari Plan ensued, calling for the supervised independence of Kosovo.

Kosovo Albanians went ahead and declared independence on February 17, 2008 with the support of major Western powers: the United States and the majority of EU member states. The EU's “supervision” of Kosovo ended 4 years later. To date, 116 states have recognized Kosovo as an independent state, while Serbia, with strong support from Russia, continues to oppose Kosovo's independence. Kosovo has been unable to secure a seat in the UN. The continued contestation of Kosovo's independence keeps the Albanian national struggle alive, and it defines the political environment in which claims for post‐conflict transitional justice are made. The issue of gender‐just peace is integral to those efforts.

Studies of nationalism and memory in the context of Kosovo's postwar transition have highlighted the role of historically entrenched, traditional values in processes of social change (Ströhle, 2010). Schwandner‐Sievers (2013) argued that international efforts at identity building cannot compete with these values, often expressed at the level of the everyday. Identity building and remembrance in Kosovo, both local and international, are entangled with nationalist discourses (Visoka, 2016), which reinforce patriarchy (Krasniqi, 2007; Perritt, 2008). Efforts dealing with sexual violence in Kosovo have struggled to disentangle gender from ethnicity and have often appealed to local culture, as well as to international norms, for support (Di Lellio, 2016). We lack an understanding of why local and everyday responses to wartime sexual violence (are unable to) challenge dominant gender hierarchies in the process of nation building.

In postwar Kosovo, wartime sexual violence has remained a deeply sensitive issue. The commission of wartime sexual violence was written out of the master narrative and collective memory of the 1998–1999 Kosovo war in the immediate postwar period. Stigma associated with wartime rape “overshadow(s) survivors” (Amnesty International Report, 2018, p. 324). Women survivors have by and large kept silent about their suffering. The *Thinking of You* art installation by artist Alketa Xhafa‐Mripa attempted to confront the stigma.

## 5 AIRING THE DRESS: THE *THINKING OF YOU* PUBLIC ART INSTALLATION

The installation took place on June 12, 2015, coinciding with the Kosovo Liberation Day, when the NATO intervention ended Serbian rule in Kosovo. This piece of public art was performed by hanging some 5,000 skirts and dresses from washing lines in the main football stadium in Kosovo's capital, Prishtina. The Kosovo‐born artist Alketa Xhafa‐Mripa and the producer of the installation, the United States‐based academic Anna Di Lellio, travelled around Kosovo to collect skirts and dresses, which were donated by members of the public, as well as local elites. Skirts and dresses were chosen as small, everyday symbols of femininity that could easily be donated by both women and men. They also highlighted the universalist goals of the installation (BBC, 2016). The project was supported, both personally and institutionally, by the president of Kosovo at the time, Atifete Jahjaga. She served as the third president of Kosovo from 2011 to 2016 and was the first nonpartisan candidate and the youngest https://en.wikipedia.org/wiki/List_of_elected_and_appointed_female_heads_of_state in Southeast Europe. She came to power following a political crisis^2^ and emerged as a consensus candidate supported by ideologically opposed political parties in Kosovo. Empowered by the political role, she took upon herself to confront the gender injustice surrounding wartime sexual violence in Kosovo. Her involvement marked a significant change in the approach to the issue in Kosovo that had previously been almost exclusively championed by civil society.

Other than donating a dress herself and promoting the installation (as well as the subsequent documentary), Jahjaga also provided support to the project through the National Council on Survivors of Sexual Violence, which she established in 2014. The project also attracted international support from high‐profile political figures, from Cherie Blair (wife of former British Prime Minister Tony Blair) and Lady Anelay (at the time, the United Kingdom's special representative on preventing sexual violence in conflict) to Western entertainment stars (for example, musician Rita Ora and actress Eliza Dushku). Xhafa‐Mripa dedicated the installation to Kosovo's survivors of sexual violence. The installation referred specifically to sexual and gender‐based violence in Kosovo, but the artist believed it spoke a universal, visual language that could be understood by everyone (Dickinson, 2016). The stated motivation behind the project was to achieve recognition of the issue since rape victims were being stigmatized in the Albanian society. Members of the public and political elites helped hang skirts and dresses, which was seen as a way to encourage the discussion of private issues more openly by “airing dirty laundry in public” (Women's Views on News, 2015). The collection of skirts, the promotion of the installation, its preparation (the hanging of the skirts), and the day of the installation were featured in a documentary film *Mendoj Për Ty|Thinking of You*–Documentary produced by Anna di Lellio and Fitim Shala.

The skirts installation should be seen as one of a number of public activities leading to a degree of recognition of wartime rape in Kosovo. As a project that received great international publicity, it overshadowed earlier local initiatives aimed at confronting the stigma and seeking justice for sexual violence, such as the Kosovo Women's Network protests, which marked International Women's Day and demanded justice for survivors of wartime sexual violence on March 8, 2012. Kosovo has since made some formal progress in the recognition of wartime sexual violence (Chick, 2016; Ristic, 2017). The amendments to the law on war victims included women survivors of war rape, who are now entitled to reparations (Kosovo Assembly, 2014). This legislative breakthrough also has gendered dynamics. Only a handful of women have applied for reparations so far and whether more will overcome the stigma and claim their rights is yet to be seen. Additionally, the range of entitlements, including the payout, for wartime rape victims is much less than those for other recognized victims of war. Women have thus been positioned as an “Other” within a nation and its hierarchy of suffering during the conflict. Consequently, women survivors of wartime sexual violence have found it difficult to reveal what they experienced, out of fear of being ostracized by families and communities (Halili, 2017). As Haxhiaj (2017) has observed, “stuck in the prison of stigma, victims see no freedom.” Art is a powerful form of storytelling with the potential to counter stigmatization by invoking empathy through identification. Excluded within the nationalist discourse, it is in art that stories of women survivors of sexual violence found a place. One example is the film *Tri Dritare dhe Një Varje* (*Three Windows and a Hanging*) directed by Isa Qosja (2014), which speaks against stigmatization and gender inequality in everyday life in Kosovo. A further example, Fëllanzat (*Blackbirds*) by Gazmend Bërlajolli (2017), is a short story based on actual testimonies by women. In this study, we are interested in the power of public art to confront stigma and acknowledge victims.

## 6 THE SYMBOL, DISCOURSE, AND PERFORMANCE: RECOGNITION THROUGH ART IN KOSOVO

We analyze the *Thinking of You* art installation to reveal how Kosovo is constructed as a nation at an everyday level through the interplay of performance, discourse, and symbols of the artistic practice. The everyday nationalism perspective allows us to capture the mundane elements of this interplay, in order to reveal how gender relations are reordered within nation building. The analysis reveals everyday enactments of gendered nationhood and recognizes the role played by formal actors and processes as well.

### 6.1 Private and public symbolism of the dress

The *Thinking of You* art installation centers on the object of a dress. The creation of the visual archive of dresses in the *Thinking of You* project uses gender as a medium through which war trauma and privations in post‐conflict Kosovo are acted out. It creates a symbolic landscape of the body mediated by an “affective economy” (Ahmed, 2004, p. 8), of nationalism as a patriarchal ideology. To the artist, “the skirt symbolizes femininity, beauty, fragility … the woman, basically” (Xhafa‐Mripa, quoted in Marí, 2015). The dress as the foundation of the artistic project can also be problematized.

The art project and its central symbol—the dress—build and reinforce essentialist understandings of gender and femininity: they objectify women. This objectification draws on the patriarchal imagining of women and femininity as “beautiful and fragile.” The dress also reinforces the sexual representation of women and confines women's subjectivities to the patriarchal framework. This dynamic is evident when the use of the dress in the *Thinking of You* art installation is put in the context of women's struggle for equality in Kosovo. The Women's Movement in Kosovo has on previous occasions built their campaigns on the dress to counteract gender‐based stereotypes as underlying causes of violence against women. A case in point is the song *Fundi im i shkurtë* (*My Short Skirt*), which the Kosovo Women's Network made central in its awareness campaign on violence against women (Corrin, 2004, p. 82).^3^ The *Thinking of You* art installation is the extension of this gendered iconography, which is the dominant code, almost shorthand, for speaking about wartime sexual violence in Kosovo. However, while the idea of a “short skirt” could be taken as subversive, almost provocative in the face of traditional mores, the role of the skirt in the *Thinking of You* art installation is more literal: the skirt positions a woman within traditional gendered boundaries.

The symbolism of the skirt as a private object is further embedded in the public symbolism of urban iconography through the staging of the art installation at the football stadium in Kosovo's capital city. The football stadium is a symbol of power. It is a predominantly male social space and as such it is gendered; it contributes to the reproduction of traditional gender roles through the persistence of the dominant position of masculinity in culture, politics, and everyday life. In the context of the art installation, the football stadium acquires another symbolic reference to the norms and ideas of postwar justice, while the gendered relationship between masculinity and femininity is visualized on its grounds through politicized women's bodies. Thus, the location of the art exhibition was a part of the message.

The football stadium is packed full of symbolic potential for the representation of the nation through interplay of masculinity and femininity in this particular space. When asked why the football stadium was chosen to showcase the project, the artist replied that it is:

a macho territory ( … ) It's a man's world; it's also a place for the sweat, the whistling, the rushing of adrenaline (…) Here the survivors, women can stand and reaffirm their existence, and that we are all one. I believe that when these women were tortured, they were enclosed in a (not necessarily physical square), they didn't see a way out (…). (Xhafa‐Mripa, quoted in Marí, 2015)

The location of the art installation in a football stadium also highlights the role of sport in the continuing and everyday reproduction of national identity (Seippel, 2017, p. 45). Specifically, it reflects and plays with the idea of the centrality of sport in Kosovo's nation building. Soon after independence, the Kosovo state applied for membership in the International Federation of Association Football (FIFA) and was rejected. Only in 2016 was the Kosovo football team accepted into FIFA, which was a highly symbolic and political moment in the realization of Kosovo's statehood and “equality with other nations” (Zeqiri, 2016). FIFA membership was seen as an achievement of Kosovo state building within the internationalization of sport. In this sense, the art installation connected not just with the sport but also, through its artistic advocacy, with the international norm of accountability for wartime sexual and gender‐based violence within the UN's Women, Peace, and Security Agenda. The resonance of this message with international audiences resulted in replications on a smaller scale of the *Thinking of You* installation in, among other places, Brussels and London (KOHAnet, 2018). The international currency that the exhibition created through the powerful imagery of skirts and dresses on the clothes line is also demonstrated by the communicative power of the image, for example, being adopted to adorn the cover of Swaine's monograph on gender and transformative justice (Swaine, 2018).

From the perspective of the mundane, the act of hanging the dresses is part of an implicit nationalist ideology in which they serve as “reminders of possibility of sacrifice” (Billig, 1995, p. 175) in honour of the nation. The national(ist) content in the symbolic communicative message projected by the dresses on the clothes lines in the football stadium is purposeful. As Atifete Jahjaga pointed out, the football stadium was used “to show the magnitude of the wartime sexual violence” (Jahjaga, 2016; Tran, 2015). This quality may move us to envision the scale of sexual violence in the 1998–1999 Kosovo war. However, by simultaneously creating proximity and distance, detachment, and closeness, the *Thinking of You* art installation also produces alienated subjectivities. The art installation leaves the gender hierarchy intact by reinforcing traditional notions of femininity, embodied by the dress; and traditional notions of masculinity, embodied by the football stadium. The project does offer women a possibility of liberation from the confines of the nation that stratifies gender relations either in war or in peacetime. Nonetheless, the gendering process has not affected the powerful, and arguably successful, internationalization of the message, even though it elides the agency of women survivors in its recognition of wartime rape.

Consequently, this symbolism constructs gender and power through binary oppositions, giving masculinity agency and rendering femininity powerless. At the same time, it seems to reaffirm the women's place in a nation: separate from men, brought to the men's space, and recognized on men's terms. In so far as the football stadium was to show physically and metaphorically how these women were trapped and how there was no escape from their suffering, the message blurred the agency of the one entrapping: was it Albanian society entrapping the women by stigmatizing them or the Serbian perpetrators doing so to commit the crime? While this ambiguity may be intrinsic to art and artistic expression, it effectively problematizes different roles of men from the two nations involved in conflict but it does not empower women victims.

### 6.2 Discourses and silences: Dress as a proxy

The recognition of wartime sexual violence interacts with dominant post‐conflict national(ist) discourse in Kosovo. Its incorporation into nation building is challenging owing to its potential to destabilize the notion of male honour central to the construction of Albanian national identity. The gendered discourses of war construct men as “righteous warriors” associated with courage and heroism, whereas women are described as “beautiful souls,” hence “non‐violent, offering succor and compassion” (Elshtain, 1987, pp. 3–13). This is the case in Kosovo, where the dominant Albanian national discourse pertaining to history, remembrance, commemoration, and hero worship from the recent conflict gives primacy to men. Male values and masculine norms based on a master narrative founded on the conflict shape the processes of “national imagining” (Di Lellio & Schwandner‐Sievers, 2006; Krasniqi, 2016). Conflict‐related gendering of the Albanian nation further reinforces the traditional equating of the Albanian nation with men (Mertus, 1996, p. 270), while it reaffirms the traditional roles of women in the service of the nation. The prevailing symbolic role of women is that of the revered mother and nurturer of the nation. Women are not viewed as actors or subjects in their own right: they are valued through their role in the domain of the family, at the expense of a larger role in society and politics. While Kosovo Albanian women have been active in the processes of nation building, the understanding of that role has been adapted to the prevailing narrative defined by celebration of masculinity (Krasniqi, 2014).

Paradoxically, the *Thinking of You* installation reproduces the subordination of women within the Kosovo Albanian nation‐building project at the same time as it attempts to counter it. Alongside the visual dimension of the skirts and dresses on the line, this project constructs a discourse that draws its authority by continually quoting from the dominant and patriarchal discourse of Albanian victimhood, victory, and heroism. It attempts to unite the male‐dominated national discourse, based on notions of men, warriors, fatherhood, heroes, and honour, and the discourse of women victims, which is constructed in oppositional terms, based on notions of women, victims, motherhood, heroines, and denigration. Within this framework, men are the leaders and creators of the nation, while women are in their service, although both are targets of the Serbian violence. The stated aim of the installation was national unity: “being as one, not them and us” (Xhafa‐Mripa, quoted in Marí, 2015). While the artist remains silent on the question of how this unity can be achieved beyond the installation itself, the discourse around the project clearly positions the recognition of a woman within the dominant patriarchal discourse of victimhood, victory, and heroism in Kosovo. By contrast, the unity of Albanians against the Serbian “enemy” is more explicitly drawn out through the shared suffering of men and women at the hands of Serbian forces. Xhafa‐Mripa states:

I just wanted to let the survivors know that we were all thinking of them. They should never feel ashamed, never their fault. Firstly, the shame lies with the Serbia occupation and then secondly with our society—there was no institution to support them. (Xhafa‐Mripa, quoted in Cela, 2017)

Referencing the dominant discourse of nationalism in order to amplify the voices of survivors contradicts the universality of the project's message. Gender‐based violence is no longer constructed as a universal problem. Instead, it is part of the struggle of the Albanian nation for independence and related to the construction of the national “Other”, Serbs. With this kind of engagement with the nation‐building project, the *Thinking of You* art installation recognizes women but only to the extent that this recognition is aligned with the dominant understanding of the nation, which is profoundly gendered. This dynamic characterizes the art project: it was launched on Kosovo Liberation Day and seen as a method to “internationalize and visualize the enormity of the crime committed by Serbian forces in Kosovo” (Garentina Kraja, Advisor on Foreign Policy and Security, interviewed in *Mendoj Për Ty|Thinking of You*–Documentary, 2016).

Holding the installation in the Prishtina City Stadium also plays into this dynamic. Silence is broken by presenting wartime sexual violence in a public and masculine arena, as discussed above. Breaking of silence is accompanied by discursively constructing women as male counterparts in a heroic national struggle. Hence, the installation aims “to break the stigma, and gain support for this group of women, the survivors, not as victims, but as heroines” (Flora Macula, UN Women, interviewed in *Mendoj Për Ty|Thinking of You*–Documentary, 2016). Instead of recognizing the victimhood of the victims of rape, the discourse engages with existing notions of national heroism, defined by dominant patriarchal mores, which remain unchanged. The discursive politics of the dress becomes complicit in constructing an obedient femininity that whispers and/or speaks through proxy. The incorporation into the dominant nation‐building project is effective: it legitimizes the art installation, without necessarily giving voice and subjectivity to women survivors of wartime rape. Survivors “would donate a dress, and quietly whisper (…), ‘I am one of them,’ and just leave” (an international expert, quoted in Zaba, 2016). Besides the voices of women survivors of sexual violence, the voices of men survivors and of survivors from other ethnic groups were silenced too. By naturalizing heterosexuality and acknowledging the victimhood of one national group, the *Thinking of You* art installation also produces and consumes gender and ethnicity while reproducing inequalities and exclusions. As commentator and civil society activist Flaka Surroi remarked, the “Thinking of You project should have been entitled ‘Acting for You’” (Surroi, 2015, p. 10).

The recognition of wartime rape is thus implicated in the reproduction of the dominant discourse through patriarchal norms and values that do not disrupt the gendered status quo. As a consequence, alternative discourses are silenced; women's primary, expected, and imagined role remains that of motherhood. The *Mendoj Për Ty*|*Thinking of You* documentary is replete with references to mothers and children of Albania. For example, the documentary ends with a rape victim describing the art installation as “a cool breeze to us, the Albanian mothers” (*Mendoj Për Ty|Thinking of You*–Documentary, 2016). The notion of motherhood is linked to nationhood in a gendered construction of the nation.

The donation of dresses also generates social relationships that extend beyond the life of the art project. Then president Atifete Jahjaga (2016) remarked that women victims “spoke through their donations.” Yet in this political economy of exchange, no attention seems to have been paid to the significance of donating; it is not a mere gesture of giving away an object. “(T)o give something is to give a part of oneself,” argued (Mauss 1998; quoted in Hamber & Wilson, 2002). The prescribed subordinate gender role within the nation remains unchanged: women's role is to *give* to the nation.

### 6.3 Performance of everyday nationhood: Waving dresses, waving flags

Political construction of nationalism in postwar and post‐independence Kosovo goes hand in hand with the construction of gender hierarchies. The relationship between gender and nation building is a product of the history of socialism in Kosovo and of the 1998–1999 Kosovo war. The collective memory of the war is manifested through myriad symbols including memorials, statues, and commemorations (Di Lellio & Schwandner‐Sievers, 2006). They are reminders of the past, deployed daily to serve the purpose of legitimatizing the Kosovo state. McClintock argues:

Nationalism takes shape through the visible, ritual organization of fetish objects—flags, uniforms, airplane logos, maps, anthems, national flowers, national cuisines and architectures as well as through the organization of collective fetish spectacle—in team sports, military displays, mass rallies, the myriad forms of popular culture and so on. (McClintock, 1995, pp. 374–375)

The *Thinking of You* art installation is a stage where everyday nationalism is played out in a temporal and spatial sense. The launch of this artistic project took take place on June 12, 2015, the 16th anniversary of NATO troops entering Kosovo, which all Kosovo Albanians regard as the day of liberation from Serbian rule. By scrutinizing this moment, we gain an insight into how nationhood is constructed through everyday practices and rituals. As Fox and Miller‐Driss (2008, p. 550) put it, national holidays “are produced and performed to induce and reproduce national solidarities.”

In the *Thinking of You* art installation, the individual and the collective exist in parallel. The individual memory of wartime sexual violence long maintained in the private domain challenges the public memory. But the experiences of survivors and their suffering become “meaningful by associating them with the teleology of the liberation struggle” (Hamber & Wilson, 2002). This association was explicitly expressed in the statements by the artist and the then Kosovo president. According to the artist:

In the beginning, it was just a day, and then it was proposed to link it with the anniversary of Kosovo's liberation. I hope it will also be the day of these women's liberation from guilt, from shame, so they can be proud out there and not be ashamed because of what happened: a liberation within themselves. (Xhafa‐Mripa, quoted in Marí, 2015)

President Jahjaga reiterated this message and further connected the experience of survivors to that of the national liberation struggle and of shared suffering. She stated that:

On the anniversary of liberation, with the survivors of torture that still live with war inside them, we shall be together. We remember the deep suffering caused by war. Our peace will never be complete without their peace. (Atifete Jahjaga speech at the launch of the *Thinking of You* art installation, quoted in Maxharaj, 2015, p. 4)

The representation of wartime sexual violence in the *Thinking of You* art installation and its subsequent political consumption point to the installation's political function. The *Thinking of You* art installation calls for national and international solidarity. It strives to be inclusive—the project is captioned in three languages: Albanian, English, and Serbian. However, following Billig's account of banal nationalism, it could be argued that waving dresses at the football stadium is akin to waving flags, which implicitly reproduces dominant nationalist ideology.

## 7 CONCLUSION

This study focuses on quotidian yet complex dynamics of gender relations from the perspective of everyday nationalism. While the concept of nationalism, including everyday nationalism, often prioritizes the study of the boundaries between ethnic “Others,” we have focused on internal boundaries drawn between female and male members of a nation. We studied the moment in time when a nation, in this case the Kosovan nation, achieved independence, considering that when the national goal of independence is achieved, women often find themselves excluded from the public sphere and power (Yuval‐Davis & Werbner, 1999, p. 1). Men regain and consolidate their power in both the private, that is, family; and the public, that is, political, leadership domains. By engaging with the transitional justice scholarship, we investigated whether subordination of women can be challenged through an artistic practice that promotes acknowledgement for wartime sexual violence. Observing everyday enactments of gender and nationhood, we show how dominant constructions of the nation are reproduced and gender asymmetries within it. Key to this dynamic is the consideration—from the bottom–up perspective—of how formal institutional frameworks come to frame the informal and intimate world of ordinary individuals, thereby limiting their agency. We found that women's trauma is recognized through its alignment with the sacrifice for the national cause. Nationalism is able to reconcile a tension between the coldness of social organizations and the emotionality that exists in microsolidarity at the everyday level (Malešević, 2013, p. 15). The art installation bolsters microsolidarity in nation building. Nationalist ideology portrays the nation as a community of close friends or a giant extended family. By attaching the public art installation to this image, the artistic practice becomes subsumed within patriarchal understandings of both the nation and family. The everyday perspective reveals how nationalism becomes entrenched in this context. Engaging with the nation‐building project in Kosovo legitimizes the public recognition of the legacy of wartime sexual violence, but it also embeds that recognition within the dominant construction of the nation. The installation's message was disseminated through existing nationalist organizational and ideological channels and became attached to the nation‐building project (cf. Malešević, 2013, p. 130). This results in recognition of the crime of sexual and gender‐based violence but not of the individual survivors.

As Das notes, wartime rape resides “between public knowledge and public secret” (Das in Mookherjee, 2015, p. ix). The *Thinking of You* art installation is also an undertaking in public memory. It contributes to public recognition that wartime rape should not be a taboo, it can be a topic of discussion and a cause for activism. The artistic intervention and its reception and appropriation in the local environment, as the everyday nationalism perspective allows us to reveal, how the exhibition serves to further naturalize the broader social/political/economic/cultural discourses and practices along binary and hierarchical gender identities and relations. The masculine conceptualization of a nation is retained in a newly reconstituted national unity that recognizes the fact of wartime sexual violence as women's sacrifice for the national cause. Everyday nationalism is the site of reproduction and legitimation of dominant discourses, symbols, and practices of gendered hierarchies instead of being a site of resistance and social transformation. The recognition of wartime sexual violence against women underpins the process of “Othering” vis‐à‐vis the ethnic “Other” but in a way that existing gender hierarchies within a nation are reproduced. Consequently, externalizing pain and seeking closure through art may contribute to public acknowledgement of difficult truths and recognition of harm and trauma. However, such interventions need to be reflective of how they may be implicated in the construction of a nation defined by patriarchal norms and values that leave no space for gender‐transformative politics.

## ENDNOTES

1 See “Who's afraid of banal nationalism? A symposium” in *Nations and Nationalism, 2018*, Vol. 24, Part 4.

2 Her appointment resolved the deadlock after president Fatmir Sejdiu of the Democratic League of Kosovo resigned and the Constitutional Court ruled against the election of Behxhet Pacolli of the New Kosovo Alliance as the president of Kosovo.

3 The Women's Movement in Kosovo emerged as an informal collective of women's groups in 1996. Since then, it has developed into a multiethnic network of 141 women's organizations from Kosovo who advocate the promotion and protection of rights and interests of women and girls in Kosovo.
